# Fluorescence Spectroscopy and Chemometric Modeling for Bioprocess Monitoring

**DOI:** 10.3390/s150510271

**Published:** 2015-04-30

**Authors:** Saskia M. Faassen, Bernd Hitzmann

**Affiliations:** Process Analytics and Cereal Science, Institute of Food Science and Biotechnology, University Hohenheim, Garbenstraße 23, 70599 Stuttgart, Germany; E-Mail: Saskia.faassen@uni-hohenheim.de

**Keywords:** fluorescence spectroscopy, chemometric modeling, sensors, biotechnology

## Abstract

On-line sensors for the detection of crucial process parameters are desirable for the monitoring, control and automation of processes in the biotechnology, food and pharma industry. Fluorescence spectroscopy as a highly developed and non-invasive technique that enables the on-line measurements of substrate and product concentrations or the identification of characteristic process states. During a cultivation process significant changes occur in the fluorescence spectra. By means of chemometric modeling, prediction models can be calculated and applied for process supervision and control to provide increased quality and the productivity of bioprocesses. A range of applications for different microorganisms and analytes has been proposed during the last years. This contribution provides an overview of different analysis methods for the measured fluorescence spectra and the model-building chemometric methods used for various microbial cultivations. Most of these processes are observed using the BioView^®^ Sensor, thanks to its robustness and insensitivity to adverse process conditions. Beyond that, the PLS-method is the most frequently used chemometric method for the calculation of process models and prediction of process variables.

## 1. Introduction

On-line measurements of substrate, products, intermediate products and other physicochemical process variables during bioreactor cultivation are becoming increasingly important. For the process analytical technology (PAT) initiative of the US Food and Drug Administration (FDA) and for quality by design (QbD) approaches, software sensors support the ambition to establish on-line monitoring methods to ensure high quality of manufacture of pharmaceutical products and batch-to-batch reproducibility [1,2,3,4].

For the on-line monitoring of critical bioprocess variables in bioreactors software sensors based on off-gas analyzers, dissolved oxygen (DO) or pH-electrodes are used. Because of the complexity of the biological matrix, the access to important process variables is limited or the sensors are not robust enough for the required conditions in bioreactors [1]. Beyond that, the application of on-line measurements, for example on-line HPLC, allows for the measurement of substrate and product concentrations during cultivation [1,5]. One disadvantage of on-line HPLC is the time delay between sampling and determination of the concentration of the observed process variable. As an alternative to on-line HPLC, the control of bioprocesses by using FIA measurements is described in [6,7], but there is also a time delay for the detection of the concentration of certain process variables. A frequently announced improvement of the detection of crucial process states can be achieved by using sensitive on-line software sensors in combination with mechanistic models for the estimation of process variables [8,9,10]. Therefore, the combination of soft-sensors with multivariate data analysis enables process supervision and control [3,11]. Recently, NIR-spectroscopy has been used for bioprocess monitoring [12,13,14], as well as Raman spectroscopy [15,16]. Both are techniques based on vibrational effects. In small molecule applications chemical compounds can be identified better using Raman than NIR-spectroscopy, but NIR-spectroscopy prevails for bioprocess fingerprinting [17]. However, both methods are not as sensitive compared to fluorescence-spectroscopy.

For more than 30 years fluorescence sensors have been applied for the monitoring of various biological processes. In biotechnology, pharma and food process engineering they are used for biomass and product prediction, process or media characterization. Fluorescence spectroscopy enables a highly developed and non-invasive technique the on-line monitoring and supervision of these processes. The maintenance of optimal process parameters is ensured by this approach. In 1970 Harrison and Chance already reported the use of the fluorescence technique for the monitoring of continuous cultures of microorganisms by recording the intensity of light emitted by reduced nicotinamide adenine dinucleotide (NADH), where a single wavelength combination of one excitation and one emission wavelength was measured [18]. What has also been known for decades is the linear correlation of culture fluorescence and the biomass concentration [19,20]. The fluorescence method is improved further by using more than only one single excitation and emission wavelength pair. For this, the fluorescence of a culture broth can be measured by a range of excitation wavelengths and a single emission wavelength or *vice versa* a single excitation wavelength and an emission spectrum or using an excitation-emission matrix (EEM) that consists of different combinations of excitation and emission wavelengths. Nowadays, EEM fluorescence spectroscopy or so-called 2-dimensional (2D-) fluorescence spectroscopy has been widely established. Here, a combination of multiple excitation and emission wavelengths is taken to observe different biological processes [21]. During microbial cultivation there are observable significant changes within the 2D-fluorescence spectra caused by variations in the concentration of biogenic fluorophores, such as aromatic amino acids, vitamins and co-enzymes [22].

A 2D-fluorescence spectrum consists of a high number of intensity values because of the different excitation and emission wavelength combinations. This leads to large data sets that require methods for data reduction and evaluation. The important process variables are only accessible by complex analysis methods that are able to detect the information hidden in the fluorescence spectra. Common approaches to get this information out of the 2D-fluorescence spectra are chemometric models, such as multiple linear regression (MLR), principal component regression (PCR) and partial least square regression (PLS) [23,24]. In addition to these linear methods, there are a number of applications of non-linear techniques, including, for example, artificial neural networks (ANN) and further machine learning methods [9,25,26].

In addition, there are a lot of attempts to reduce the high number of variables by selecting the wavelengths combinations out of the whole 2D-spectra that are important for the description and estimation of certain process variables. For this task also artificial intelligence algorithms containing for example self-organizing maps, genetic algorithm and ant colony algorithm are described [9,27,28,29,30].

The reduction of the large data sets by using an ant colony optimization (ACO)-based methodology is reported for the analysis of near infrared (NIR) spectra [31]. This method is also applicable for the evaluation and wavelength selection of 2D-fluorescence spectra. 

There are a high number of different attempts for the usage of fluorescence spectroscopy. Following, the fluorescence spectroscopy is set up for different types of cultivations of microorganisms and feeding procedures in the biotechnology, pharma and food process engineering. In this contribution, recent applications with its evaluation methods are discussed. 

**Table 1:** Applications of fluorescence spectroscopy for cultivation processes.

Organism	Type	Cultivation	Fluorescence	Reference
*Escherichia coli*	Bacteria	Batch, Fed-Batch Continuous	2D-Fluorescence, NAD(P)H fluorescence	[9,27,32,33,34,35,36,37,38,39,40]
*Wautersia eutropha*		Batch	NAD(P)H fluorescence	[41]
*Bacillus polymyxa*		Batch	2D-Fluorescence	[42]
*Klebsiella pneumonia*		Batch	2D-Fluorescence	[43]
*Aspergillus oryzae*		Batch, Fed-Batch	2D-Fluorescence	[44]
*Alcaligenes eutrophus*		Fed-Batch	2D-Fluorescence	[25]
*Aspergillus niger*		Fed-Batch	2D-Fluorescence	[45]
*Pseudomonas aeruginosa*		Batch	2D-Fluorescence	[38,46]
*Azohydromonas australica*		Batch	NAD(P)H fluorescence	[20]
*Bacillus*		Fed-Batch	2D-Fluorescence	[47]
*Streptomyces coelicolor*		Fed-Batch, Continuous	2D-Fluorescence	[28,29]
*Pichia pastoris*	Fungi	Batch	2D-Fluorescence	[48,49,50,51]
*Saccharomyces cerevisiae*		Batch, Fed-Batch	2D-Fluorescence	[10,11,27,37,52,53,54,55]
*Claviceps purpurea Hansenula polymorpha*		Batch Batch	2D-Fluorescence NAD(P)H fluorescence	[8,40]
*NSO Cells*	Mammalian	Batch	2D-Fluorescence	[50]
*Baby Hamster Kidney Cells*		Batch, Fed-Batch	2D-Fluorescence	[56]
*Chinese Hamster Ovar Cells*		Batch, Fed-Batch	2D-Fluorescence	[30,57,58]
*Azadirachta indica*	Plant	Batch	NAD(P)H fluorescence	[59]
*Eschscholtzia California*		Batch	2D-Fluorescence	[60]
*Catharantuhus roseus*		Batch	2D-Fluorescence	[60]

## 2. Fluorescence Spectroscopy 

### 2.1. Principles and Fluorophores

Sensors based on fluorescence spectroscopy are widely used for different applications in bioprocess monitoring (Table 1). However, just three of these cultivation processes [26,43,54] are carried out on an industrial scale. All other applications were only executed on a laboratory scale.

Fluorescence spectra can be measured *in situ* and on-line in real-time. The principle of this measurement method is the interaction of light and matter. For this reason, a fast and non-invasive measurement technique is used as a requirement for on-line and real-time supervision and control for bioprocesses [61].

**Figure 1:**
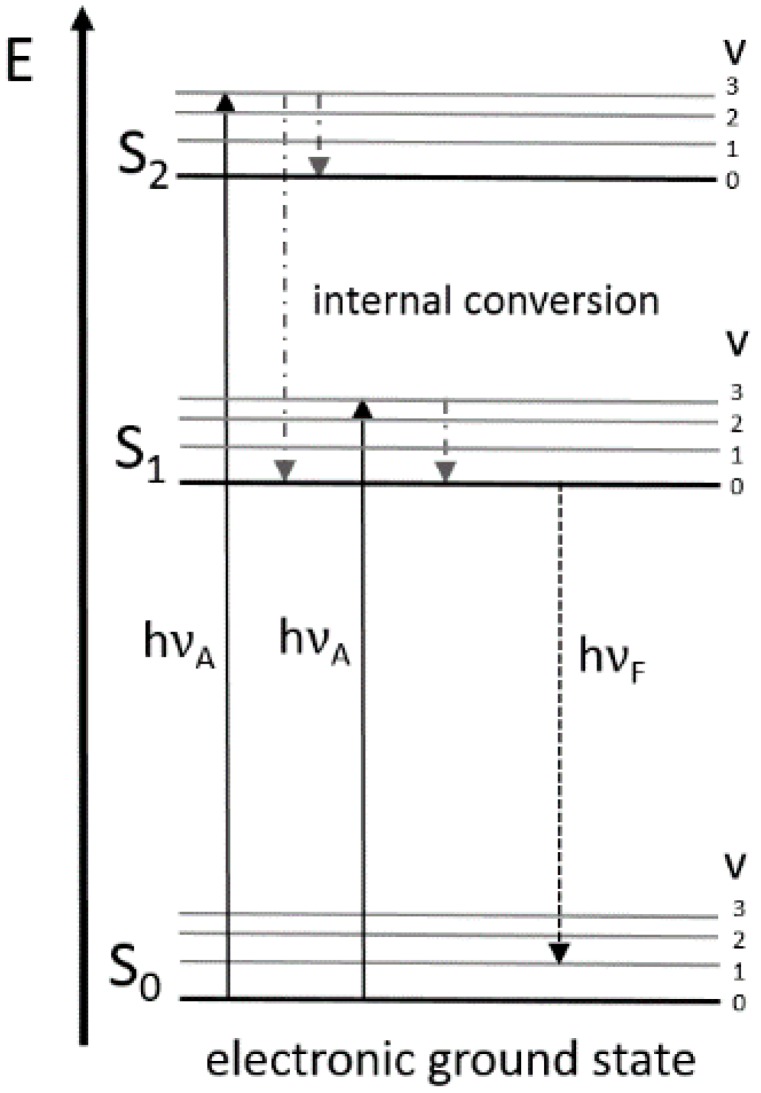
Energy level diagram (Jablonski diagram) for visualization of fluorescence phenomena (see explanation in the text).

The fluorescent activity of the analyte occurs from certain molecules that emitted light after absorption. These so-called fluorophores contain an aromatic system. The emission wavelength is greater than the excitation wavelength, caused by an energy loss. The process of the fluorescence is illustrated by the Jablonski diagram shown in Figure 1.

**Figure 2:**
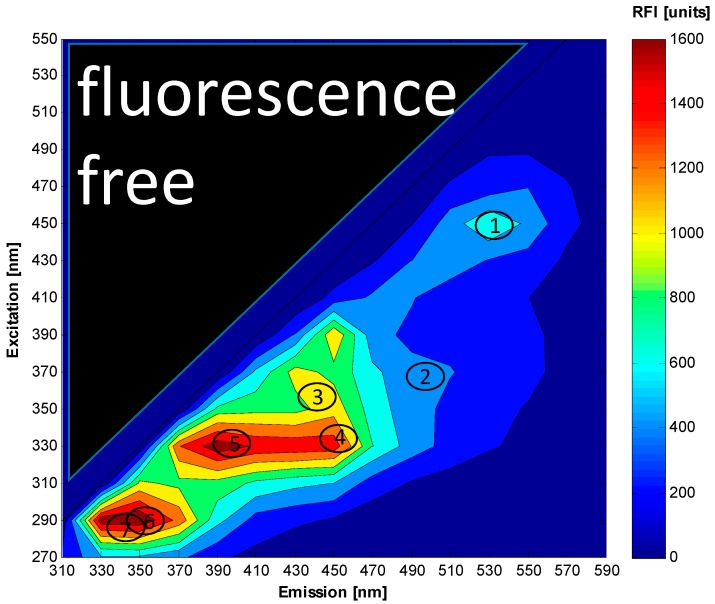
Contour plot of an excitation-emission matrix measured with the BioView^®^ sensor from *S. cerevisiae* cultivation with fluorescence maxima of flavin (1); riboflavin (2); NADH (3); NADPH (4); pyrodoxin (5); tryptophan (6) and tyrosine (7).

A light quantum of energy *hv_A_* supplied by an external source is absorbed by the fluorophore, creating an excited electronic singlet state (S_1_ or S_2_). On these energy levels the fluorophores can exist in different vibrational energy levels (v) corresponding to the Franck-Condon-principle. From the higher vibrational level of S_1_ or S_2_ the fluorophore is rapidly relaxing to the lowest vibrational level due to internal conversion. A photon with energy *hv_F_* is emitted when the fluorophore is returning to its electronic ground state S_0_. The energy of this emission photon is lower, and therefore of a longer wavelength than the excitation photon. This behavior can be seen in Figure 2, where in the upper left triangle of the spectrum no fluorescence signal can be seen. This so-called Stoke’s shift enables the high sensitivity of the fluorescence technique because it allows the detection of emission photons against a low background, isolated from excitation photons [62]. Characteristic features of fluorophores are the quantum yield and the lifetime. The ratio of the number of excited and emitted photons is the quantum yield, and the lifetime is defined by the average time the molecule spends in the excited state before it returns to its ground state [63]. However, the fluorescence yield can be influenced by different effects which involve energy transfer and absorption. For example, the so-called inner filter effects reduce the intensity of the fluorescence measurements when non-fluorescent components of the medium absorb excitation or emission radiation while reducing the fluorescence yield of an observed fluorophore [64]. The excited state fluorescence lifetime also changes with changes in the environment. Furthermore, the culture fluorescence depends on bioprocess variables, such as the optical density (OD), viscosity, pH, the aeration of the bioreactor and a lot of further the fluorescence measurements affecting variables [22].

In biotechnological processes the fluorescence measurements are based on fluorescent proteins and biological molecules which show endogenous fluorescence. Examples of fluorescent proteins and molecules with biogenic fluorescence are presented In Table 2 molecules that show biogenic fluorescence include the amino acids and vitamins as well as the co-enzymes flavin adenine dinucleotide (FAD), NADH and reduced nicotineamide dinucleotide phosphate NAD(P)H [63].

**Table 2:** Excitation and emission wavelengths for some fluorophores used in biotechnology.

Fluorophore		Max Excitation Wavelength (nm)	Max Emission Wavelength (nm)	Reference
GFP	fluorescence proteins	400, 470	505, 540	[62]
EYFP		514	527	[62]
mCherry		587	610	[65]
Tryptophan	amino acids	280, 290	350	[63,66]
Tyrosine		275/278	280, 300/330–350	[63,66,67]
Phenylalanine		260	280, 282	[63,68]
FAD, Flavins	co-enzymes	450	535	[63]
NADH		290, 351	440, 460	[63]
NAD(P)H		336	464	[63]
Pyrodoxin	vitamins	332, 340	400	[63]
Vitamin A		327	510	[63]
Riboflavin		365	520	[66]

The typical excitation ranges from the ultraviolet to the visible range of electromagnetic waves and the red shifted emission light spans the ultraviolet and visible spectral range. By applying 2D-fluorescence technique to cultivations of microorganisms one is able to detect changes in the spectra occurring during the cultivation caused by biogenic fluorophores (Figure 2).

### 2.2. Fluorescence Spectrometer

Further development of early fluorescence sensors, based on only a single wavelength combination for monitoring NADH or tryptophan emission [18,61], establish the 2D-fluorescence sensors as a commonly used method. Table 3 presents different fluorescence sensors and their specified measuring range. 

**Table 3:** Fluorescence sensors applied for bioprocess monitoring.

Type	Wavelength Selector	Wavelength	Resolution	Reference
BioView^®^	Filter	Excitation: 260–560 nm Emission: 300–600 nm	20 nm	[8,9,10,11,28,29,32,33,42,43,44,47,48,50,51,52,53,57,60,69]
FLUOstar^®^	Filter	NADH Signal	-	[34,39,40]
Hitachi F4500	Grating	Excitation 200–890 nm Emission 200–900 nm	10 nm	[11,27,35,36,37,45,46]
Perkin Elmer LS 50 B /55	Grating	Excitation: 200–800 nm Emission: 200–650/900 nm	1 nm	[26,49,56]
Varian Cary Eclipse	Grating	up to 900 nm	1.5 nm	[25,30]
Varian VIPL 3120	Filter	NADH Signal	-	[41,59]
Ingold Type Fluorosensor	Filter	Excitation: 360 nm Emission: 450 nm	-	[20]
USB2000 spectrometer	Grating	200-1100 nm	10 nm	[55]
FL3095	Grating	Excitation: 260–680 nm Emission: 320–950 nm	-	[54]

**Figure 3:**
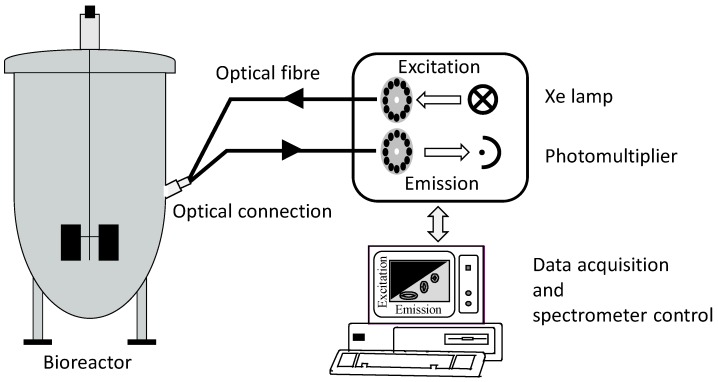
Overview of the BioView^®^ set up.

A small number of research groups still uses fluorescence sensors to monitor only the NADH signal. The BioView^®^ sensor has clearly turned out to be the most frequently employed fluorescence spectrometer. The wavelength selection is done by filter systems or by monochromators when grating technology is used. The BioView^®^ sensor selects the different wavelength combinations by using two filter wheels (Figure 3).

Each filter wheel has 16 slots for adding filters of specific emission and excitation wavelengths as well as no filter for the measurement of scattered light. These filter wheels can be controlled individually. The default setting of the step size is 20 nm. The fluorescence spectrometer is combined with the bioprocess via optical light guides. The measuring time for the scanning of a complete 2D-fluorescence spectrum taking all filters is roughly 1 min, which allows almost continuous measurements, resulting in large data sets for a whole fermentation. However, not all of the data contain relevant information about the process. The analysis and evaluation of bioprocesses by using continuous fluorescence measurements are done by different chemometric methods, such as the principal component analysis (PCA), partial least squares regression (PLS) or neural networks (NN). These methods filter the significant information out of the data sets.

## 3. Extracting Information Out of Fluorescence Spectra

The fluorescence spectra contain a lot of useful information about the observed biological processes. The extraction of this information is done by different approaches. In general, the following processing steps have to be done: the first step during evaluation is a preprocessing step that in some way normalizes, centers or filters the raw data to avoid effects such as noise or differences caused by different intensity maxima of the fluorescence values. The preprocessing is followed by a data reduction, decomposition or wavelength selection step, where large data sets are reduced by transforming the data or by using just a selection of all variables of the data set. Afterwards a chemometric model is calculated according to the measured process variables and the preprocessed fluorescence spectra. The quality of the models is assessed with different evaluation methods. Chemometric models of a process can then be used for the monitoring and supervision of these processes.

### 3.1. Preprocessing

As also typical of other measurement techniques, measurement noise can mask the information hidden in a spectrum. Furthermore, the information is not necessarily only contained in the high intensity values in the spectra but may be in the low shoulders intensity values too. Hence, to avoid these effects a preprocessing is recommendable. The batch-to-batch variability can be reduced by calculating a difference spectrum. The first spectrum after inoculation or an average of three to five spectra from the beginning of the cultivation is subtracted from all following spectra to get the difference spectra [8,9,10]. Therefore, just the changes in the fluorescence intensity values during a fermentation are considered. Additionally, to reduce the influence of noise the fluorescence signals can be smoothened by using the average values over a few spectra [42,69] or using the Savitzky-Golay-filter [70]. A subsequent normalization is performed on a spectrum by dividing each value by the spectra average. Beside this, one can divide each data point in a spectrum by the corresponding data point in the first spectrum which will generate a kind of normalized spectrum [56]. As the last preprocessing step, the data can be centered and weighted to unit variance as well as normalized. The normalization can be done by using different methods. For fluorescence data some of the methods used are, for example, the normalization of the data by dividing each value by the spectrum average value or by subtracting the spectrum mean from each value followed by a division of the standard deviation of the spectrum, which is called SNV-transformation [71]. All these preprocessing steps enhance the quality of the continuous evaluation and analysis of fluorescence data. However, there is no common rule indicating which preprocessing procedure is the best one.

### 3.2. Data Reduction, Decomposition and Wavelength Selection

Various types of methods for data reduction, selection of relevant wavelength combination and data decomposition are recommended for the evaluation of fluorescence spectra from biotechnological processes (Table 4).

Using 2D-fluorescence spectra leads to large data sets caused by the simultaneous measurements of different excitation and emission wavelengths. One spectrum consists of a high number of fluorescence intensities, but not all of them contain important information according to the requested process variable. Different strategies are known to handle this problem. Commonly used are methods that transform the data to variables with high variance and variables with low variance, containing just background noise of the measurement, such as the principal component analysis (PCA) [72]. The high dimensional data sets of the multivariate data are reduced using PCA.

The original data matrix *X* decomposes into the product of two smaller matrices—the score T and loading matrix P—plus a residual matrix E, containing just noise.

(1) $$X=TP^{t}+E$$

**Table 4:** Methods for data reduction, decomposition and wavelength selection.

Method	Application	Software	Reference
PCA	Data evaluation, Data reduction	Unscrambler^®^, MATLAB^®^	[10,25,26,29,33,42,56,69]
SWR	Wavelength selection	MATLAB^®^	[53,55]
MROBPCA	Data quality and outlier detection	MATLAB^®^	[58]
MCR-ALS	Data decomposition	Unscrambler^®^, MATLAB^®^	[30,54]
SIMPLISMA	Data decomposition	Unscrambler^®^, MATLAB^®^	[54]
SOM	Data reduction, Classify spectra	MATLAB^®^ ViscoverySOMine	[9,27,35,36,37]
PARAFAC	Data decomposition, Data evaluation/selection	MATLAB^®^	[10,29,47,48]
GA	Wavelength selection	MATLAB^®^	[28,29,53]
ES	Wavelength selection	MATLAB^®^	[53]
iPLS	Wavelength selection	MATLAB^®^	[28]
PV	Wavelength selection	MATLAB^®^	[28]
ACO	Wavelength selection	MATLAB^®^	[30]
CARS	Wavelength selection	MATLAB^®^	[30]

The score matrix T provides information about the actual state of the process, while the loading matrix *P* includes information regarding the extraction of knowledge from the intensity values of a spectrum. The original data matrix is transformed into a new matrix with principal components (PC), sorted by their variance. Most of the variance is contained in the first PC, in the second there is less variance than in the first and more variance than in the third and so on. By taking the first principal components, holding almost the whole variance of the data, the dimension of the original data matrix *X* is reduced dramatically. Because often one, two or three PCs contain approximately all the variance of the data—the others representing only noise—the data can be visualized by plotting a so-called score plot, where further interpretations become possible, for example the identification of different process states. 

Parallel factor analysis (PARAFAC) decomposes data into high important factors and areas by taking the emission-excitation-matrix in its original three-way array structure [73,74,75]. As already described for the PCA, the PARAFAC transforms the data array into sets of loading matrices and a residual matrix by mostly reducing the dimension of the data. The PARAFAC models are a straightforward extension from the two-way PCA to multi-way data. The fluorescence data is arranged in a three-way array (measurement time × excitation wavelength × emission wavelength). The PARAFAC model can be described with the following equation, where F is the number of PARAFAC components which are considered here:

(2) $$x_{ijk}=\overset{F}{\underset{f=1}{\sum }}a_{if}b_{jf}c_{kf}+e_{ijk}$$

*x_ijk_* is the intensity of *i*th spectrum at the *j*th emission wavelength and at the *k*th excitation wavelength. The contribution of the spectrum to each component is represented by the parameters *a_if_*, *b_jf_*, *c_kf_*, and the residuals *e_ijf_* containing the noise. The values of *a_if_*, *b_jf_*, and *c_kf_* are calculated by minimizing the sum of squared *e_ijf_*. The spectra are decomposed into *F* PARAFAC components which represent the concentrations of hypothetical substances. For fluorescence excitation-emission data the loadings constructed out of *a_if_*, also referred as scores, may be interpreted as the relative concentration of process variable *f* in sample *i*, the loading vector of *b_if_* elements as the estimated emission spectrum and the *c_if_* loading vector is the estimated excitation spectrum of this process variable.

**Figure 4:**
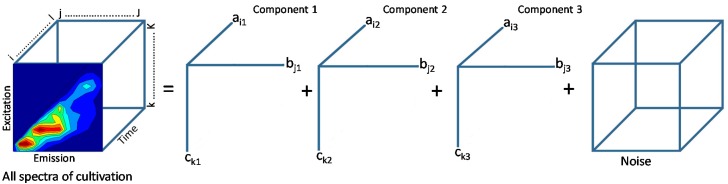
PARAFAC model with three components (F = 3) for the modeling of three process variables.

As presented in Figure 4 the components of the PARAFAC model have a direct chemical interpretation, e.g. concentration of certain process variables, in a valid model. In the case of fluorescence spectra the single components with their emission and excitation loadings correspond to certain fluorophores. The scores of the model are estimates of the relative concentrations of the fluorophores identified by the loadings [76]. Different fluorophores are described by the calculated components and can be used as a reduced data set for further analysis. The PARAFAC analysis results in a model with a reduced set of variables to a few components model that describes almost the whole variance of the data set. One obvious advantage of the PARAFAC method is the unique solution of the model. An estimated PARAFAC model cannot be rotated without a loss of fit. Both PARAFAC and PCA belong to linear decomposition methods. PARAFAC decomposition is more robust, because it uses less parameters than PCA decomposition. For some examples PCA models might exist, however no PARAFAC model.

**Figure 5:**
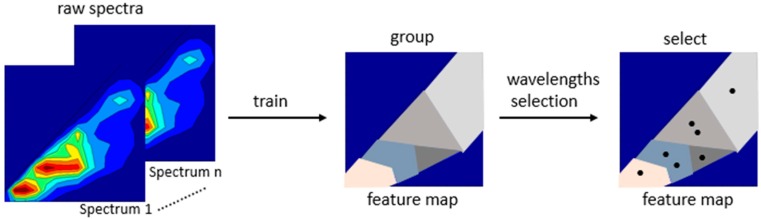
Illustrated overview using SOM for fluorescence spectra.

In contrast to this, the self-organizing map (SOM) is an unsupervised neural network approach for data decomposition [77]. By using SOM fluorescence data can be classified without any external supervision. This non-linear algorithm enables the mapping of the three-way fluorescence spectra with identifying those combinations of excitation and emission spectra with useful information of the process variables. The SOM projects high-dimensional data onto lower dimensional data, onto a structured set of neurons, while retaining the data topology [77]. The neural network consists of only two layers, an input and an output (SOM) layer. Therefore, the 2D-fluorescence spectrum is transformed from the matrix format into one-dimensional vectors following normalization. Every element in the input vector is connected to every neuron in the output layer. The weight vectors *w_j_* of the feature map have the same dimension as the input vector *x_i_*. The neurons of the feature map compete for the input with their internal parameters. The neuron with the nearest matching parameters wins. For every iteration during the training of the SOM, a distance of d(*x_i_*, *w_i_*) is calculated and used for their measures of similarity where different distance metrics can be used, for example the Euclidean distance. The closest neuron to the input vector is chosen with its corresponding weight vector. The results of the SOM are classified spectral data presented by a feature map where the topological relationships hidden in the large input data sets are retained (Figure 5) [27,37]. Afterwards, a wavelength selection while reducing the whole data set can be done by choosing representative data of each of those groups. For example, by the calculation of Euclidean distances within each class, representative wavelength combinations are found.

The optimal number of classes in a given feature map can be determined by estimating the degree of scattering of the fluorescence intensities of all spectral components in the corresponding class by computing the time-dependent variance of fluorescence intensities of all of the spectral components in the class [27]. The large data set containing all combinations of excitation and emission wavelengths can be reduced by selecting just a few wavelengths containing the process information by using SOM.

Other methods for the selecting of single wavelength combinations are machine learning methods inspired by Nature, such as evolutionary algorithms like the genetic algorithm (GA) [78,79] or swarm algorithms, including the ant-colony algorithm (ACO) [80]. Both algorithms mimic principles of Nature to find a subset of wavelength combinations that are able to describe the observed biological process. Further data decomposition and wavelength selection methods are described elsewhere (see Table 4). The decomposition of the large data set or the reduction by wavelength selection can improve the models while decreasing the computational effort.

### 3.3. Modeling for Variable Prediction 

With the help of fluorescence spectroscopy measurements, meaningful information about the physiological state of a microorganism or process states can be obtained. Thus, to convert the information contained in the fluorescence spectra, modeling approaches must be applied. As an input to the modeling procedure the reduced data are used (such as the principal components of PCA, the PARAFAC components and the selected wavelength combinations of SOM). The procedure of calibrating a model and applying it for prediction is performed in two steps. Starting with the on-line measurements of 2D-fluoresscence spectra$\;x_{i}\;$and the corresponding off-line values $y_{i}$ of the process variables of interest from one or more initial cultivations, a process model will be calculated (step one). Afterwards, the values of the process variables $y_{i}$ of a new cultivation can be predicted from the 2D-fluorescence spectra using the calculated model (step two).

A lot of different approaches are described in literature, including linear and non-linear methods as well as supervised and non-supervised learning procedures (see Table 5). Beside the different methods for the calculation of the chemometric models, there are a lot of various evaluation techniques for the prediction of quality. The models are calculated by using the whole 2D-fluorescence spectra or by taking reduced data as described above (Section 3.2). Most of the models for monitoring microbial cultivations are based on linear modeling, especially the partial least squares (PLS) regression method [23,75,81]. PLS enables the identification of the factors that not only show the largest amount of variance, but also allows a linear correlation between the fluorescence spectra and process variables via the covariance of the two data sets. Besides PLS, some other linear techniques are mentioned, including principal component regression (PCR) [36,69], linear weighted regression (LWR) [20,28,41,49,59] or multi-linear method, such as n-way partial least squares regression (NPLS) [10,28,47,58]*.* All these methods build models by identifying at least bi-linear correlations between the process variables and the fluorescence spectra [82,83].

Because of the inherently nonlinear nature of biological processes, the applications of different kinds of artificial neural networks (ANN) are described by some research groups [35,69,84], using different architectures. Different algorithms are implemented to handle cultivation data with neural networks [85,86], for example using feed-forward neural networks (FFNN) [21] with back-propagation neural networks (BPNN) [31]. By applying these models important bioprocess variables can be predicted on-line and in real-time, such as biomass, substrates and products as can be seen in Table 5. There is just one example where a closed loop for substrate feeding is described [10]. They implemented a control algorithm by predicting the metabolic state of the yeast cells by using fluorescence spectra. With this control procedure the yield of biomass was much higher due to a pure oxidative growth of the cells. For the implementation of the models different software is used. As can be seen in Table 5, Matlab^®^ has most of all applications. Different special toolboxes for building chemometric modeling are available in Matlab^®^, for example the PLS-toolbox or the neural-network-toolbox. Altogether, there is a broad range of different applications for fluorescence spectroscopy, chemometric models, implementation software and various validation techniques (details in Table 5).

When a process model is used for the prediction of process variables, the quality of that prediction must be judged. The evaluation of the predicted values ${\hat{y}}_{i}\;$can be done by using, for example, the root mean square error of prediction (RMSEP), or the goodness of the fitted model can be assessed by using the coefficient of determination (*R*^2^).

(3) $$RMSEP=\sqrt{\frac{1}{n}\overset{n}{\underset{i=1}{\sum }}(y_{i}-{\hat{y}}_{i}}{)}^{2}$$

(4) $$R^{2}=1-\;\frac{\sum_{\text{i}=1}^{\text{n}}\left({\text{y}}_{\text{i}}-{\hat{\text{y}}}_{\text{i}}\right)}{\sum_{\text{i}=1}^{\text{n}}\left({\text{y}}_{\text{i}}-{\bar{\text{y}}}_{\text{i}}\right)}$$

## 4. Conclusions and Future Trends

The application of fluorescence spectroscopy, especially 2D-fluoresence, is becoming more and more interesting for the analysis of processes in the biotechnology, food and pharma industry. This fact is also pushed by PAT and QbD efforts. The batch-to-batch reproducibility, the control and automation get improved by using chemometric models based on fluorescence spectroscopy. The fast analysis enables the identification of critical process states in time and increases the efficiency of biotechnological processes.

**Table 5:** Process models and applications.

Method	Application	Evaluation	Software	Ref.
PLS	Glycoprotein yield prediction	Relative errors: 2.3%–4.6%	MATLAB^®^	[30]
	Glycerol/methanol prediction	Mean prediction errors: 7%–10%	Unscrambler^®^	[51]
	Biomass/polymixin prediction	RMSECV: biomass 0.4 g/L, polymixin 35 mg/L	Unscrambler^®^	[42]
	Biomass, glucose, ethanol and product prediction	R^2^: biomass 0.53, glucose: 0.88	MATLAB^®^	[55]
	OD, glycerol and 1,3-propanediol prediction	ethanol 0.01, product 0.73		
	Biomass, glucose, CPR	RMSEP: OD 0.78 units,	MATLAB^®^	[43]
		glycerol 10 g/L, 1,3-PD 2.6 g/L		
	Cell density and glycoprotein	RMSEP: biomass (three conditions) 3.9%–40.7%, glucose 6.8%, CPR 9.1%	Unscrambler^®^	[33]
		in 95% confidence interval, *R*^2^ = 0.91 cell density, 0.99 glycoprotein		
	Biomass and glycerol	RMSEP: biomass 0.67/0.729 glycerol 1.52/0.911	-	[56]
	Total amino acids, biomass	RMSECV: CDW 1.02 g/L , AA 1.06 g/L		
	Cell count (CC), OD, po2%	RMSECV: CC 1.029, OD 0.046, pO2% 5.358 R2: CC 0.936, OD 0.988, pO2% 0.977	MATLAB^®^	[48]
		RMSEP: ALA 38.512 mg/L DO 5.1506%	MATLAB^®^	[28]
	Extracellular 5-aminolevulinic acid (ALA), disolved oxygen (DO), CO_2_	CO_2_ 0.756%	MATLAB^®^	[54]
	Biomass, protein, alkaloid	RMSEP: biomass 7.26%, proteins 5.74%,	Unscrambler^®^	
		alkaloids 3.37%	MATLAB^®^	[36]
	Glucose, lactate, glutamine	RMSEP: glucose 0.524 g/L, lactate 0.494 g/L		
		glutamate 0.0155 g/L *R*^2^: glucose 0.967,	Unscrambler^®^	[8]
		lactate 0.972, glutamate 0.983		
	Cellmass, lipase activity	*R*^2^: cellmass 0.73–0.97, lipase activity 0.93	Unscrambler^®^	[57]
		RMSECV cellmass 0.77–1.48 g/kg		
	Biomass	RMSEP: 4.6 g/L		
	Biomass, ethanol, glucose	RMSEP: 4%, 2%–8%, 4%	MATLAB^®^	[44]
	Regulation of optimal feed	-		
	Biomass, glucose	-	MATLAB^®^	[29]
	Biomass	RMSEP: 0.19 g/L (PLS),	MATLAB^®^	[10]
	pH-value, acidity	RMSEP: 2.36%–4.84%, 6.04%–8.08%	Unscrambler^®^	[11]
	Enzyme activity	RMSEP: 0.08–0.12	MATLAB^®^	[25]
			MATLAB^®^	[52]
			MATLAB^®^	[69]
			MATLAB^®^	[47]
PCA	Plasmid containing strain stability	-	SIMCA-P 8.0	[32]
	Medium wash steps, cell growth	-	Mathematica	[46]
	Cultivation description with scores	-	MATLAB^®^	[36,37]
PCR	Extracellular 5-aminolevulinic acid (ALA), disolved oxygen (DO), CO_2_	RMSEP: ALA 38.344 mg/L DO 5.296%	MATLAB^®^	[36]
		CO_2_ 1.225%		
	pH-value, acidity	RMSEP: 3.60%–5.10%, 6.45%–9.97%	MATLAB^®^	[69]
Linear regression Linear regression	Biomass prediction	*R*^2^ = 0.9869	-	[41]
	Biomass and PHB prediction	linear correlation to NADH signal	-	[20]
	Biomass	MARE = 0.12	MATLAB^®^	[49]
	Biomass	*R*^2^ = 0.91	-	[59]
	Total amino acids, biomass	RMSECV: CDW 1.18 g/L, AA 0.80 g/L	MATLAB^®^	[28]
NPLS	Estimation of product yield	RMSEV: 0.13 g/L	MATLAB^®^	[58]
	Enzyme activity	RMSEP: 0.08–0.12	MATLAB^®^	[47]
	Total amino acids, biomass	RMSECV: CDW 1.39 g/L, AA 2.17 g/L	MATLAB^®^	[28]
	Biomass	RMSEP: 5%–7%	MATLAB^®^	[10]
PARAFAC	Cultivation description	-	MATLAB^®^	[44]
	Biomass	RMSEP: 0.20 g/L	MATLAB^®^	[52]
Luedeking-Piret-based equation	Biomass	MARE = 0.06	MATLAB^®^	[49]
ANN	3-Chloro-4-methylaniline	*R*^2^ > 0.7	microCortex	[26]
	pH value, acidity	RMSEP: 2.44%–3.42% , 6.89–12.11	MATLAB^®^	[69]
FFNN	Biomass, glucose	R^2^: glucose 0.88, biomass 0.93 Largest observed error: biomass 1 g/L, glucose 8 g/L	MATLAB^®^	[25]
BPNN	Biomass, glucose, CO_2_, DO, O_2_,	evaluation of BPNN topology all R_xy_ > 0.97	MATLAB^®^	[35]
	Total amino acids	RMSEP 0.112–0.165 g/L		
RBF	Biomass (BDM), total cell number (TCN), dead cells (DC), product, plasmid copy number (PCN)	BDM 0.5 g/L, TCN 17 1/mL, DC 1%	MATLAB^®^	[9]
		Product 7 mg/g BDM, PCN 8 units		

For this, the monitoring and prediction of the biomass concentration is the most described process variable. The applicability of fluorescence sensors for microbial cultivation, especially for *E. coli* and *S. cerevisiae* cultivations, is reported by a lot of research groups (Table 1). In addition there are further applications to more microorganisms and fungi as well as plant and mammalian cell lines. The comparison of the results, published by different research groups, is hindered by the different established quality criterions. The RMSEP is sometimes given in relative concentration or in percent. However, it is not mentioned what the reference is, the maximum or the average of each individual value of the observed process variable, which can make a big difference. In other cases the R^2^ is provided, but without any related estimation errors for the evaluation of the prediction quality. Standardized quality criterions are desirable for further investigations so that results can be compared.

Nevertheless, all these approaches demonstrate that the process monitoring and automation can be highly improved by using fluorescence spectroscopy in combination with chemometric modeling. As evaluation technique PLS methods are dominating. However, also other approaches including ANN, GA and ACO show great potential to increase the analysis quality of fluorescence-based process control. While analyzing fluorescence-based data it should be taken into account that medium fluorescence or other effects as the mentioned inner filter or cascade effects can reduce the applications of fluorescence sensors. The quality and performance of processes in biotechnology, food and pharma industry can be carried out with methods for quality control, such as the “golden batch”, where an optimal batch becomes the starting point for all following batches. An ideal process control strategy can be implemented with the help of process knowledge from using fluorescence sensors and process models. Altogether, the establishment of process sensors based on fluorescence spectroscopy should continue for further improvement of biotechnical processes in the future.

Off all published applications of fluorescence spectroscopy for bioprocess monitoring just three came out of an industrial environment, indicating that there is a big gap between the academic (laboratory scale) and the industrial application. One reason for this might be the fact that just for new implemented processes new measurement methods have a chance to be installed. Therefore, there will be always a kind of lag-phase between academic and industrial applications. But this lag-phase can be long because, as Max Planck mentioned [87]: “A new scientific truth does not triumph by convincing its opponents and making them see the light, but rather because its opponents eventually die, and a new generation grows up that is familiar with it.” However, another reason for the gap might be the overall costs, which include the investment costs, the (re-)calibration cost as well as the maintenance cost. Although no reagents are necessary, the measurement method is mostly indirect. Furthermore, today just multipurpose fluorescence spectrometer are around. A fluorescence sensor which uses just specific wavelength combinations for a specific application will be of low-cost for the hardware. Therefore, using such sensors fluorescence applications even in an industrial environment might increase in the future.
